# Mast Cell Interaction with Neutrophils in Human Gastric Carcinomas: Ultrastructural Observations

**DOI:** 10.1155/2016/6891971

**Published:** 2016-11-02

**Authors:** Antonio Ieni, Valeria Barresi, Giovanni Branca, Rosario Alberto Caruso, Giovanni Tuccari

**Affiliations:** Department of Human Pathology “Gaetano Barresi”, University of Messina and Azienda Ospedaliera Universitaria “Policlinico Gaetano Martino”, 98125 Messina, Italy

## Abstract

*Aim.* The role of mast cells in cell-cell immune interactions has received increasing attention, although their functional interaction with neutrophils still remains to be clarified in tumors. The aim of the present study was to investigate the association between mast cells and neutrophils in a series of gastric carcinomas (GC).* Patients and Methods.* 52 surgically resected GC specimens were routinely processed for both light and electron microscopy. Only cases showing both mast cells and neutrophils in the tumor stroma were considered in the analysis.* Results.* Only 9 GC (M : F = 5 : 4; age range: 50–82 years) showed both mast cells and neutrophils in the tumor stroma. At ultrathin sections, we identified heterotypic aggregation and intermingling of mast cells and neutrophils. Mast cells had mature phenotype and showed full complement of granules with homogeneous, scroll, particle, and mixed pattern. In addition, we found normal-appearing or early apoptosis showing neutrophils.* Conclusion.* Our histological findings showed the likely interaction between mast cells and neutrophils in GC. We hypothesize that the granular content of mast cells may be released in small quantity through a mechanism called “kiss-and-run fusion,” which is alternative to well-known massive anaphylactic or piecemeal degranulation.

## 1. Background

Despite its decreasing incidence and mortality, gastric cancer (GC) still represents the second leading cause of cancer death worldwide [1–3]. Approximately, 20% of cancer deaths worldwide are associated with unresolved infection or chronic inflammation; moreover, prolonged inflammation can lead to mucosal atrophy, which in some patients precedes neoplastic development [4]. In particular, unresolved inflammation may create a microenvironment facilitating cellular transformation and chronic tissue damage. In addition, it may trigger repair response including growth and survival factors, tissue-remodeling enzymes, and immune regulatory cytokines [5]. However, the role of immune cell populations in gastric cancerogenesis has not been yet fully clarified [4]. Neutrophils, mast cells, eosinophils, and dendritic cells may directly infiltrate foveolar epithelium, whereas the lamina propria is permeated by mononuclear cells, such as lymphocytes, macrophages, and plasma cells [6–10].

By virtue of their strategic position, mast cells represent a major sensory arm of the innate immune system [11, 12]. Mast cells are granulated tissue-resident cells of hematopoietic lineage which may be found near epithelium or at the intraepithelial level as well as near the vessels in the connective tissue [9, 11, 12]. Mast cells are a prominent source of proinflammatory mediators and cytokines that can induce inflammation, vascular changes, and leukocyte infiltration [11, 12]. In particular, mast cell-derived tumor necrosis factor-alpha plays an important role in inflammation through the recruitment of neutrophils in the sites of infection [13, 14]. However, mast cell degranulation is also responsible for the characteristic signs of inflammation and neutrophil infiltration around the wound site [15, 16]. Recently, mast cells have received increasing attention as emerging protagonists in cell-cell immune interaction [17–20]. Although functional interaction between mast cells and neutrophils has not been yet clarified, morphological heterotypic aggregation between these two cell populations has been described in mouse experimental studies on contact dermatitis [21]. In the present study, we report morphological interaction between mast cells and neutrophils in the stroma of human GC.

## 2. Materials and Methods

52 surgically resected GC specimens were initially considered. None of the patients had undergone preoperative irradiation or immunochemotherapy. All samples were routinely processed for both light and electron microscopy. Briefly, fresh tumor specimens were divided into two portions by using a sharp razor blade. One portion was 10% formalin-fixed, paraffin-embedded, and stained with hematoxylin and eosin (H&E). The other portion was minced into smaller pieces and immediately fixed in 3% phosphate-buffered glutaraldehyde, pH 7.4, and postfixed in 1% osmium tetroxide for electron microscopy. Semithin Giemsa-stained sections were reviewed by light microscopy to select cases showing both mast cells and neutrophils in the tumor stroma. Tissue blocks of selected cases were cut by using Ultratome III, LKB (Stockholm-Bromma, Sweden), to obtain ultrathin sections, which were double-stained with uranyl acetate and lead citrate and finally examined and photographed under a JEOL 1200 EX TEM electron microscope (JOEL, Tokyo, Japan) at 70 kV.

## 3. Results

Of the 52 GC specimens initially considered, only 9 showed both mast cells and neutrophils in the tumor stroma. Those cases included five males and four females (age ranging between 50 and 82 years). Tumor diameter ranged between two and eight cm. According to Laurèn classification, six cases were intestinal-type GC and three were diffuse-type GC [22]. All cases were at stage IIA or IIB; 6/9 were with N0 status, while three were T3N1/T2N2.

**Figure 1:**
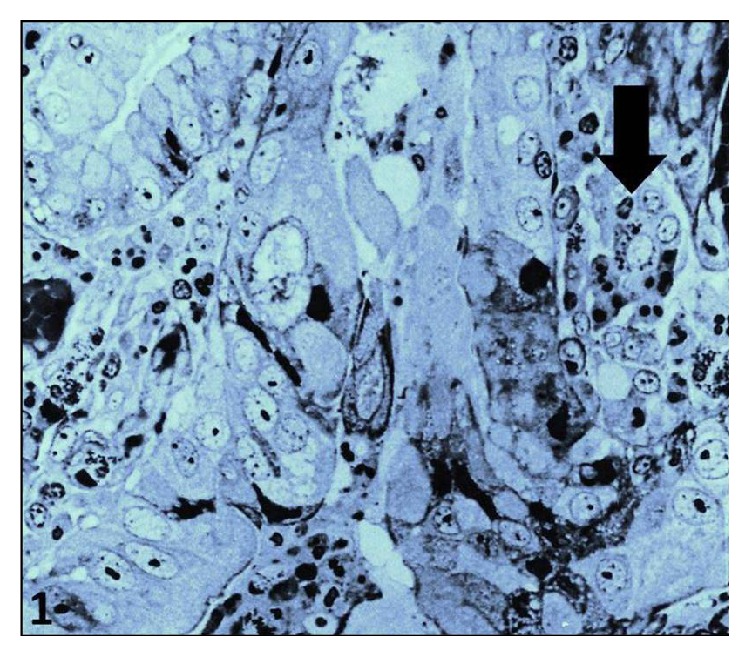
Mast cell surrounded by neutrophils (arrow) in the tumor stroma of gastric adenocarcinoma. (semithin section, ×200).

In Giemsa-stained semithin sections, mast cells were easily identified by the presence of typical granules (Figure 1, arrow). Generally, they occurred as single cells or occasionally in clusters containing one to two mast cells and one to four neutrophils (Figure 1).

**Figure 2:**
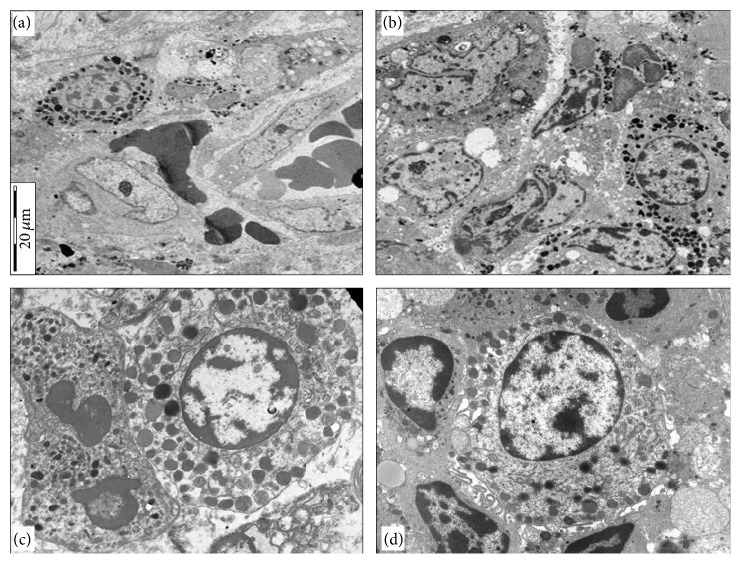
Mast cell showing a full complement of granules is in contact with a neutrophil, characterized by early apoptotic changes ((a), ×4000); another example of aggregation between mast cells and apoptotic neutrophils in the tumor stroma of a gastric adenocarcinoma ((b), ×4000); mast cell established pointed or flat areas of membrane contact with a neutrophil showing early apoptotic changes. Mast cell exhibits cytoplasmic microtubules, some of which are in close vicinity to granules ((c), ×10000); neutrophil satellitosis around mast cell showing peripheral location of granules and cytoplasmic microtubules. Some neutrophils show early apoptotic changes ((d), ×6000).

Ultrathin sections revealed the presence of heterotypic aggregation and intermingling of mast cells and neutrophils (Figures 2(a) and 2(b)). In detail, mast cells had mature phenotype with full complement of granules showing homogeneous, scroll, particle, and mixed pattern (Figures 2(a) and 2(b)). In other areas, mast cells showed peripheral location of granules (Figures 2(b) and 2(c)) and sometimes granules were localized close to the cell membrane (Figures 2(c) and 2(d)). We also found few swollen granules and numerous cytoplasmic microtubules (Figures 2(c) and 2(d)) and association of microtubules and granules (Figure 2(c)). Neutrophils located close to mast cells were morphologically well preserved or exhibited ultrastructural features of early apoptosis, such as nuclear chromatin separation into dense and electron-lucent areas, preservation of cytoplasmic granules, focal loss of glycogen particles, and preserved plasma membrane (Figures 2(a), 2(b), 2(c), and 2(d)).

## 4. Discussion

Previous experimental studies have ultrastructurally documented close spatial relationship between mast cells and neutrophils in IgE-mediated late-phase cutaneous response of mouse contact dermatitis [21]. In detail, mast cells contained nonfused swollen granules filled with altered contents and showed extrusion of membrane-free granules through membrane pores [21]; interestingly, few mast cells secreted membrane-bound granules into the dermis without cell membrane damage [21]. Successively, the number of mast cells prominently increased and total serum IgE level was greatly elevated; in addition, mast cells which showed typical anaphylactic degranulation and several immature mast cells, characterized by well-developed Golgi apparatus, were also observed [21]. To our knowledge, the above-mentioned ultrastructural features of mast cell-neutrophil interaction have not been described either in human or in experimental neoplasms thus far. Therefore, this study is the first to document the morphological relationship between mast cells and neutrophils in human GC. Nonetheless, we are not able to explain why this close cellular association occurred in only 9 of 52 GC independently from gender and age of the patients and histotype of the tumor. Nevertheless, the evidence that all cases were stage IIA/IIB may suggest the appearance of above-mentioned cellular relationship in low stage, less aggressive GC.

The morphological ultrastructural features which we observed in this series of GC can be summarized as follows: (1) whenever present, mast cells had large areas of close contact with neutrophils; (2) several neutrophils exhibited satellitosis-like pattern around mast cells; (3) mast cells close to neutrophils were functionally activated as documented by the presence of numerous cytoplasmic microtubules and fewer granules peripherally located; (4) neutrophils were well preserved or showed early apoptotic changes. However, in our previous paper, we already provided ultrastructural evidence of coupled mast cells and eosinophils in the tumor stroma of gastric carcinoma similar to that previously demonstrated in vitro through direct cell-cell adhesion [23]. Receptor/ligand couples between mast cells and eosinophils including CD48/2B4, CD226/CD112, and LFA-1/ICAM-1 were also previously described [24].

Receptors-ligand pair that may confirm the cross-talk between mast cells and neutrophils has not been reported thus far. Ultrastructurally, the analysis of degranulation in mast cells revealed two basic characteristic morphologic patterns, identified as massive anaphylactic degranulation and piecemeal degranulation [25]. The first one, the anaphylactic degranulation, is characterized by rapid secretion (within a few minutes) via degranulation channels or by direct fusion of granule containers with the cell membrane [25]. The second one, the piecemeal degranulation, presents partially or completely empty granule chambers in the absence of intergranular fusion [25]. Moreover, together with anaphylactic or piecemeal degranulation, granular contents of mast cells can be released in small portions by an alternative mechanism, called kiss-and-run fusion, involving transient opening of a fusion pore for the release of some or all of the granules without loss of mast cell integrity [26]. Therefore, although inconclusive, our ultrastructural findings in GC allow excluding mast cells anaphylactic degranulation and/or piecemeal degranulation and hypothesizing “kiss-and-run” mechanism. In fact, no evidence of massive anaphylactic type degranulation was found, while only a focal loss of granular density without fusion of intergranular membranes (piecemeal type) was encountered. Moreover, most of mast cell granules were intact and appeared to concentrate peripherally, sometimes near to plasma membrane, suggesting transient opening of a fusion pore, such as that hypothesized in the kiss-and-run fusion mechanism.

To date, degranulation of mast cells has not been linked to apoptosis of neutrophils, although mast cells were reported to induce apoptosis in vascular smooth muscle cells, endothelial cells, cardiomyocytes, and macrophages in vitro [27–30]. Both tryptase and chymase were suggested not only to be proapoptotic by themselves but also to potentiate the proapoptotic action of histamine [27]. Therefore, we can hypothesize that mast cells can firstly enhance and later suppress neutrophil activity in GC stroma. Accordingly, mast cells are responsible for the increased neutrophil infiltration during the inflammatory phase in other human pathological conditions, such as wound repair mechanism [31, 32]. Therefore, cross-talk between mast cell and neutrophils, observed in the neoplastic stroma of GC, exhibits interesting analogy with the inflammatory phase of wound healing.

## 5. Conclusion

In the present study, a close morphological association between mast cells and neutrophils was ultrastructurally documented in a series of GC. The analysis of degranulation in mast cells suggested that their granular content can be released in small portions by a mechanism called kiss-and-run fusion, which is alternative to well-known massive anaphylactic or piecemeal degranulation. The physiopathological relationship between mast cells and neutrophils in GC requires further investigation and its potential prognostic significance needs to be clarified.
